# Neuroprotective Effects of Triterpene Glycosides from *Glycine max* against Glutamate Induced Toxicity in Primary Cultured Rat Cortical Cells

**DOI:** 10.3390/ijms13089642

**Published:** 2012-08-02

**Authors:** Hyung-In Moon, Jai-Heon Lee

**Affiliations:** Department of Medicinal Biotechnology, College of Natural Resources and Life Science, Dong-A University, Busan 604-714, Korea; E-Mail: hoffmannu@nate.com

**Keywords:** *Glycine max*, glutamate-induced toxicity, neuroprotective, triterpene glycosides

## Abstract

To examine the neuroprotective effects of *Glycine max*, we tested its protection against the glutamate-induced toxicity in primary cortical cultured neurons. In order to clarify the neuroprotective mechanism(s) of this observed effect, isolation was performed to seek and identify active fractions and components. From such fractionation, two triterpene glycosides, 3-*O*-[*α*-l-rhamnopyranosyl(1–2)-β-d-glucopyranosyl(1–2)-β-d-glucuronopyranosyl]olean-12-en-3β,22β,24-triol (**1**) and 3-*O*-[β-d-glucopyranosyl(1–2)-β-d-galactopyranosyl(1–2)-β-d-glucuronopyranosyl]olean-12-en-3β,22β,24-triol (**2**) were isolated with the methanol extracts with of air-dried *Glycine max*. Among these compounds, compound **2** exhibited significant neuroprotective activities against glutamate-induced toxicity, exhibiting cell viability of about 50% at concentrations ranging from 0.1 μM to 10 μM. Therefore, the neuroprotective effect of *Glycine max* might be due to the inhibition of glutamate-induced toxicity by triterpene glycosides.

## 1. Introduction

Phytochemical compounds from grains are abundant in the human diet, particularly in fruit, vegetables and legumes, which have been consistently associated with a decreased risk of nutritional disease. They constitute one of the most abundant groups of natural metabolites and are now recognized for their important contribution to both health (and diet) effects in humans and animals. Epidemiological studies have consistently shown that regular consumption of fruits, vegetables, whole grains, and other plant foods is associated with reduced risk of developing chronic diseases, such as cancer and cardiovascular disease [1]. Legumes, especially *Glycine max*s are widely consumed in the world, and are a staple food in Korea as well as being a major source for protein, energy, vitamins, and minerals. The phytochemicals of *Glycine max*s, including phenolic compounds, phytic acid, triterpene glycosides, and phytosterols, may be responsible for their anticancer activity [2]. It was reported that *Glycine max*s reduced azoxymethaneinduced colon cancer in a rat model [3]. Legume tannins are 15–30 times more effective at quenching peroxyl radicals than simple phenolics, thus they are potential biological antioxidants [4]. Although biological activity has been discovered from *Glycine max*, it has not been fully characterized.

Alzheimer’s disease (AD) is an age-related neurodegenerative disease that affects approximately 24 million people worldwide [5]. Neuronal death is an important feature of both acute and chronic neurodegenerative diseases. AD is associated with the accumulation of l-glutamate (Glu) deposits in senile plaques and neurofibrillary tangle lesions in specific areas of brain. Glutamate, a major excitatory amino acid neurotransmitter in the central nervous system (CNS), involved in fast synaptic transmission, neuronal plasticity, outgrowth and survival, memory, learning and behavior. Glutamate also plays an important role in microglial neurotoxicity in AD. Activated microglia produce large amounts of glutamate, which induces excitotoxicity via *N*-methyl-d-aspartate (NMDA) receptor signaling [6]. Activated microglia release large amounts of glutamate through upregulation of glutaminase expression and induce excitoneurotoxicity through NMDA receptor signaling [7]. Apart from the physiological role of glutamate, excessive activation of its receptors can also evoke neuronal dysfunction and even damage or death. Glutamate-mediated neurotoxicity appears to play a crucial role in several neuropathological disorders, particularly in Alzheimer’s disease, Parkinson’s disease, epilepsy and ischemic stroke [8]. Thus, neuroprotection against glutamate-induced toxicity has been a therapeutic strategy for preventing and/or treating both acute and chronic forms of neurodegeneration [9].

In the context of our natural drugs discovery program dealing with the development of new potential neuroprotection agents, we have examined the isolation of two compounds, 3-*O*-[α-l-rhamnopyranosyl(1–2)-β-d-glucopyranosyl(1–2)-β-d-glucuronopyranosyl]olean-12-en-3β,22β,24-triol (**1**) and 3-*O*-[β-d-glucopyranosyl(1–2)-β-d-galactopyranosyl(1–2)-β-d-glucuronopyranosyl]olean-12-en-3β,22β,24-triol (**2**) as leads for novel glutamate-induced toxicity inhibitors.

## 2. Results and Discussion

**Figure 1:**
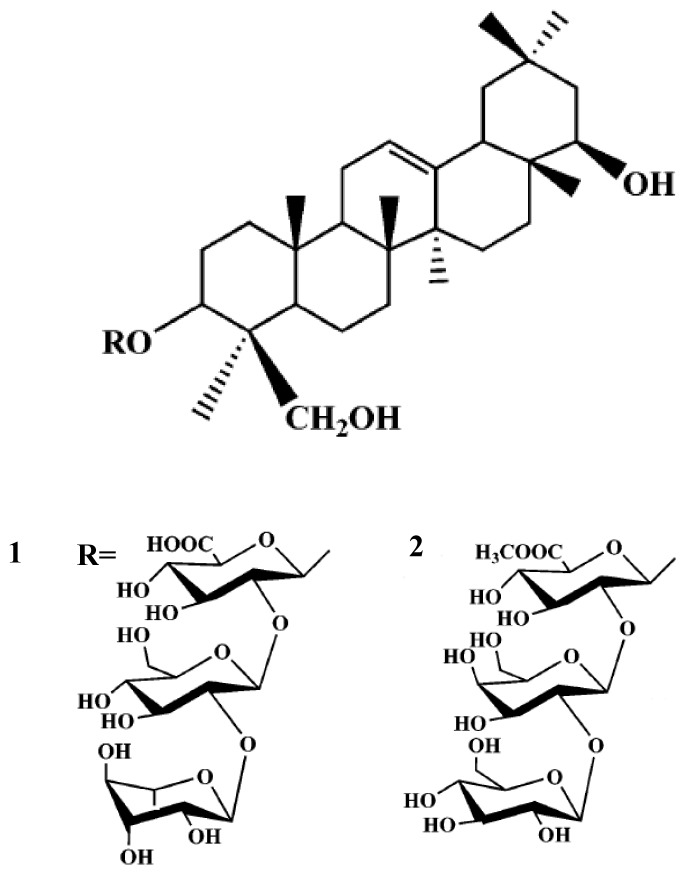
Isolated compounds structure from *Glycine max*.

It was found that the methanol extract of *Glycine max* might inhibit glutamate-induced toxicity in primary cultured rat cortical cells. In order to clarify the neuroprotective components of *Glycine max*, as a part of continued study of the neuroprotection effects of *Glycine max*, isolation was performed to seek active fractions and components. After solvent fractionation, the inhibiting effects of various fractions on neuroprotective activity were compared and the ethyl acetate fraction of *Glycine max* was found to inhibit this activity in a dose-dependent manner in the assay system using glutamate-induced toxicity in primary cultured rat cortical cells. To clarify the active compounds of *Glycine max*, the effects of the major compounds from *Glycine max* on neuroprotective activity were examined. Isolation was further performed to yield a single compound. Bioactive triterpene glycosides derivatives were isolated from the ethyl acetate fraction by repeated chromatography and recrystallization. The ethyl acetate fraction (35 g) was further purified by silica gel chromatography (230–400 mesh, 500 × 75 mm) and four subfractions (BB-1–4) whose main active subfraction (BB-3; 5.8 g) was purified by VLC and HPLC, and compound fractions 1 (7.2 mg) and 2 (4.7 mg) were obtained from hexane/ethyl acetate (20:3) elution. Then, the subfraction CH_2_Cl_2_/MeOH (10:1) was further purified on preparative HPLC using the 250 × 22 mm i.d., 5 μm, Agilent C_18_ preparative HPLC column, which was eluted isocratically with 20% acetonitrile in water at a flow rate of 8.0 mL/min. Compounds **1** (3.2 mg) and **2** (2.1 mg) were obtained at retention times of 5.3 and 14.7min, respectively. Thin layer chromatography and preparative high-performance liquid chromatography methods of the MeOH extract of harvested *Glycine max* led to the isolation of two compounds. Compound **1** was identified as 3-*O*-[α-l-rhamnopyranosyl(1–2)-β-d-glucopyranosyl(1–2)-β-d-glucuronopyranosyl]olean-12-en-3β,22β,24-triol. Compound **2** was identified as 3-*O*-[β-d-glucopyranosyl(1–2)-β-d-galactopyranosyl(1–2)-β-d-glucuronopyranosyl]olean-12-en-3β, 22β,24-triol (Figure 1). The compounds have previously been reported as antiproliferative and antioxidative activity from the *Glycine max* seed coats by Dong *et al*. [10]. In this study, we found that methanol extract of *Glycine max* might inhibit glutamate-induced toxicity in primary cultured rat cortical cells. In order to clarify the neuroprotective components of *Glycine max*, as part of a continued study of neuroprotection effects of *Glycine max*, isolation was performed to seek active fractions and components. After solvent fractionation, we compared the inhibiting effects of various fractions on neurotoxicity activity. The BB-3 fraction of *Glycine max* was found to inhibit the activity in a dose-dependent manner in glutamate-induced toxicity assay using primary cultured rat cortical cells (Table 1). To clarify the active substances of *Glycine max*, we examined the effects of the major isolated compounds from BB-3 fraction on neuroprotective activity. Isolation was further performed to yield single compounds.

Two known compounds were isolated from BB-3 fraction by repeated VLC and HPLC. To investigate and compare the neuroprotective activities of these compounds isolated from the methanol extracts of *Glycine max*, the activities of compounds **1** and **2** were evaluated in glutamate-injured primary cultured rat cortical cells at concentrations ranging from 0.1 to 10 μM (Table 2). It is notable that the neuroprotective activities of compound **2** was comparable to those of MK-801, APV and CNOX, and, only compound **2** displayed an activity which significantly attenuated glutamate-induced toxicity at concentrations ranging from 0.1 to 10 μM and exhibited cell viabilities of 50–70%.

## 3. Experimental Section

### 3.1. Plant Material

*Glycine max* cultivars (Black beans) were purchased from the local market in Hwa Sung, Kunggi-Do, Korea. The botanical identification was made by Hyung-In Moon in Dong-A University (Busan, Korea). Voucher herbarium specimens were deposited with the reference number (DA-002–003) in the Herbarium of the Department of Medicinal Biotechnology, College of Natural Resources and Life Science, Dong-A University.

### 3.2. Extraction and Isolation of Compounds

The *Glycine max*s (1 kg) were extracted by percolation in 95% methanol (MeOH; 2000 mL) at room temperature for 1 week and filtered. The combined MeOH extracts (212 g) were concentrated under reduced pressure at a temperature not exceeding 35 °C. The residue was re-percolated again. This process was repeated four times. Water (500 mL) was added and the resultant mixture successively extracted with chloroform, ethyl acetate, and *n*-butanol, respectively The IC_50_ value for a neuroprotective effect of each fraction was found only for the ethyl acetate extracts fraction. The ethyl acetate fraction which possessed the potential neuroprotective effect (IC_50_ = 81.2 μg/mL) was BB-subfraction chromatographed on silica gel using chloroform and methanol. The ethyl acetate fraction (30 g) was applied to a silica gel column and eluted with chloroform–methanol mixtures of increasing polarity (95:5 (500 mL), 9:1 (500 mL), 4:1 (500 mL), 7:3 (500 mL), 1:1 (500 mL)) to give four subfractions (BB-1–4) whose main active subfraction (BB-3; 5.8 g) was purified by VLC and HPLC. This compounds with isolate methods were modified to Dong *et al*. [8].

### 3.3. Neuroprotective Activity Testing

Primary cultures of mixed cortical cells containing both neurons and glia were prepared from 17–19-day-old fetal rats (Sprague-Dawley) as described previously [11]. Cultures were allowed to mature for at least 2 weeks before being used for experiments. Test fraction and compounds were dissolved in DMSO (final concentration in culture, 0.1%). Cortical cell cultures were washed with DMEM and incubated with test compounds for 1 h. The cultures were then exposed to 100 μM glutamate and maintained for 24 h. After the incubation, the cultures were assessed for the extent of neuronal damage by measuring the efflux of LDH (lactic dehydrogenase) which reflects the integrity of cellular membrane. The results are expressed as means ± standard errors (S.E.). The data were statistically analyzed by one way ANOVA. Differences with *p* < 0.05 were considered significant.

## 4. Conclusions

Glutamate-induced neurotoxicity has been implicated in the neuronal cell death of neurological disorders such as ischemia, trauma, seizures, and Alzheimer’s disease [6]. During our search for potential natural products against glutamate-induced neurotoxicity, we have discovered that the ethyl acetate sub-fraction (BB-3 fraction) of *Glycine max* showed significant protective activity. The neuroprotective activities of the triterpene glycosides glycosides were evaluated by the MTT assay measuring the viability in primary cultured rat cortical cells after glutamate insult. Among these two triterpene glycosides glycosides, compound **2** significantly attenuated neuronal cell death induced by glutamate in cultures. It was found to exhibit the most potent neuroprotective activity at a concentration of 10 μM. Our result supported that compound **2** was isolated from the *Glycine max* possess neuroprotective activities at low concentration and in a dose-dependent manner in primary cultured rat cortical cells. At present, the cellular and molecular mechanisms that underlie the action of compound **2** are not fully understood. However, future research data will show whether compound **2** acts on glutamate receptors, especially NMDA receptors by the following proposed pathway: (i) a protection of cortical cultures, (ii) selective protection in NMDA-induced neurotoxicity; (iii) blocking Ca^2+^ influx in glutamate insult; (iv) a reduction in NO and cellular peroxide production; (v) a reduction in GSH depletion and membrane lipid peroxidation. On the basis of our present study, the protective effect of compound **2** from *Glycine max* may provide some ideas for preventing and/or treating the neurodegenerative diseases.

## Figures and Tables

**Table 1:** Neuroprotective effects of extract and fractions from *Glycine max* against glutamate induced toxicity in primary cultured rat cortical cells a.

Dose	Cell Viability b,d (%)		

	5 μg/mL	25 μg/mL	100 μg/mL
Control c		100	
Glutamate-treated c,e		0	
MeOH extracts	13.4 ± 0.3	38.5 ± 3.2 *	59.0 ± 3.6 **
Chloroform fraction	-	-	12.3 ± 1.3
Ethyl acetate fraction	-	21.4 ± 6.3 *	55.3 ± 5.7 **
n-butanol fraction	-	-	5.8 ± 1.3
BB-1 subfraction	-	-	-
BB-2 subfraction	-	-	-
BB-3 subfraction	15.6 ± 3.6 *	36.7 ± 3.7 *	43.5 ± 2.6 **
BB-4 subfraction	-	-	26.7 ± 8.9
BB-5 subfraction	-	-	-

a Rat cortical cell cultures were incubated with test compounds for 1 h. The cultures were then exposed to 100 μM glutamate for 24 h. After the incubation, the cultures were assessed for the extent of neuronal damage;

b Cell viability was measured by the LDH assay;

c LDH released from control (0.1% DMSO concentration in culture) and glutamate-treated cultures were 11.7 ± 1.3 and 47.9 ± 4.0 units/mL, respectively;

d Cell viability was calculated as 100 × (LDH rel*e*ased from glutamate-treated-LDH released from glutamate + test compound-treated)/(LDH released from glutamate-treated-LDH released from control). The values shown are the mean ± STD of three experiments (3–4 cultures per experiment). Results differ significantly from the glutamate-treated:

* *p* < 0.05,

** *p* < 0.01,

*** *p* < 0.001;

e Glutamate-treated value differed significantly from the untreated control at the level of *p* < 0.001.

**Table 2:** Neuroprotective effects of triterpene glycosides from *Glycine max* against glutamate induced toxicity in primary cultured rat cortical cells a.

Dose	Cell Viability b,d (%)		

	0.1 μM	1 μM	10 μM
Control c		100	
Glutamate-treated c,e		0	
**1**	14.2 ± 0.3	16.7 ± 1.2	21.4 ± 5.6
**2**	16.7 ± 1.5 *	39.2 ± 1.5 **	71.5 ± 6.8 ***
APV f	11.5 ± 1.4	26.5 ± 2.3 *	42.5 ± 3.7 *
MK-801 g	51.4 ± 4.6 **	63.5 ± 5.8 ***	78.4 ± 2.0 ***
CNQX h	23.5 ± 3.7 *	44.8 ± 3.5 *	53.2 ± 4.6 ***

a Rat cortical cell cultures were incubated with test compounds for 1 h. The cultures were then exposed to 100 μM glutamate for 24 h. After the incubation, the cultures were assessed for the extent of neuronal damage;

b Cell viability was measured by the LDH assay;

c LDH released from control (0.1% DMSO concentration in culture) and glutamate-treated cultures were 11.7 ± 1.3 and 47.9 ± 4.0 units/mL, respectively;

d Cell viability was calculated as 100 × (LDH released from glutamate-treated-LDH released from glutamate + test compound-treated)/(LDH released from glutamate-treated-LDH released from control). The values shown are the mean ± STD of three experiments (3–4 cultures per experiment). Results differ significantly from the glutamate-treated:

* *p* < 0.05,

** *p* < 0.01,

*** *p* < 0.001;

e Glutamate-treated value differed significantly from the untreated control at the level of *p* < 0.001;

f APV: dl-2-amino-5-phosphonovaleric acid, a competitive NMDA receptor antagonist;

g MK-801: dizocilpine maleate, a noncompetitive NMDA receptor antagonist;

h CNQX: 6^−^cyan°-7-nitroquinoxaline-2,3*-*dione, non*-*NMDA receptor antagonist.
